# QTL mapping of almond kernel quality traits in the F_1_ progeny of ‘Marcona’ × ‘Marinada’

**DOI:** 10.3389/fpls.2024.1504198

**Published:** 2024-11-27

**Authors:** Felipe Pérez de los Cobos, Agustí Romero, Leontina Lipan, Xavier Miarnau, Pere Arús, Iban Eduardo, Ignasi Batlle, Alejandro Calle

**Affiliations:** ^1^ Institut de Recerca i Tecnologia Agroalimentàries (IRTA), Mas Bové, Ctra. Reus-El Morell Km 3, Constantí Tarragona, Spain; ^2^ Institut de Recerca i Tecnologia Agroalimentàries (IRTA), Centre de Recerca en Agrigenòmica (CRAG), CSIC-IRTA-UAB-UB, Cerdanyola del Vallès (Bellaterra), Barcelona, Spain; ^3^ Centre for Research in Agricultural Genomics (CRAG) CSIC-IRTA-UAB-UB, Cerdanyola del Vallès (Bellaterra), Barcelona, Spain; ^4^ Grupo de Investigación Calidad y Seguridad Alimentaria, Centro de Investigación e Innovación Agroalimentaria y Agroambiental (CIAGRO-UMH), Universidad Miguel Hernández, Carretera de Beniel, Alicante, Spain; ^5^ Institut de Recerca i Tecnologia Agroalimentàries (IRTA), Fruitcentre, PCiTAL, Lleida, Spain

**Keywords:** *Prunus dulcis*, breeding, kernel traits, linkage mapping, quantitative trait loci

## Abstract

Almond breeding is increasingly focusing on kernel quality. However, unlike other agronomic traits, the genetic basis of physical and chemical kernel quality traits has been poorly investigated. To address this gap, we conducted a QTL mapping of these traits to enhance our understanding of their genetic control. We phenotyped fruit samples from an F_1_ population derived from the cross between ‘Marcona’ and ‘Marinada’ for up to four years, using conventional and image analysis methods. Additionally, the 91 individuals of the population were genotyped with the almond Axiom™ 60K SNP array, and high-density linkage maps were constructed. These analyses identified several genomic regions of breeding interest. For example, two regions on chromosome one were found to contain QTLs for kernel shape and dimension, while another region at the end of the same chromosome contained QTLs for kernel fatty acid composition. Notably, QTLs for kernel symmetry and kernel shoulder, reported for the first time in this study, were also mapped on chromosome one. These QTLs will serve as a foundation for developing molecular markers linked to kernel physical and chemical quality traits in almonds, facilitating the integration of marker-assisted selection into breeding programs.

## 1 Introduction

Almond [*Prunus dulcis* (Miller) D.A. Webb, syn. *P. amygdalus* (L.) Batsch] is the most important tree nut species worldwide, with a production steadily increasing over the last 15 years. In 2022, global in-shell almond production reached nearly 4 million tons, yielding 1.6 M tons of kernel. The U.S. accounted for the majority of the almond production (79%), followed by Australia (8%) and Spain (6%) (FAOSTAT, 2024). The crop exhibits remarkable adaptability to diverse climates and irrigation regimes (Alonso et al., 2017; Martínez-Gómez et al., 2017), ranging from the fully irrigation practiced in California to the traditional dryland farming in some Mediterranean and Asian countries (Gradziel et al., 2017). This wide adaptability highlights almond as a promising crop for addressing the challenges of climate change in various regions, where less resilient crops may prove unsuitable for cultivation.

Almond have a wide range of uses, including raw consumption, snacks, desserts, marzipans, cookies, ice creams, etc. Each application has specific quality requirements, and different almond varieties are better suited to particular uses. Thus, several kernel traits like shape, dimension, fatty acids, lipid content, vitamins, phytosterol content, minerals, proteins, carbohydrates, or fiber were described at cultivar levels to assess the quality, and attractiveness, and study their best industrial performance (reviewed in Yada et al., 2011; Romero, 2014; Kodad, 2017; Flankin and Mitchell, 2019). Differences between almond in physical traits such as kernel dimensions, shape, surface color, or ease of skin removal are well-characterized and serve as unique features for usage and marketing. Similarly, variation in nutritional composition among cultivars highlights the influence of their genetic makeup and its interaction with factors such as geographical origin, climatic environment, and growing conditions (Beltrán et al., 2021; Rabadán et al., 2018; Lipan et al., 2019).

Despite extensive research on the physico-chemical traits of almonds, information on their genetic basis remains limited. The first marker-based genetic studies focused on agronomic traits, such as self-compatibility (Ballester et al., 1998), flowering time (Ballester et al., 2001), productivity and ripening date (Sánchez-Pérez et al., 2007), using low-density linkage maps that identified several major genes and quantitative trait loci (QTLs). Subsequent genetics studies on kernel quality traits initially targeted monogenic features, such as the amygdalin content related to kernel bitterness/sweetness (Sánchez-Pérez et al., 2007, 2010). Later, genomic regions associated with the content of chemical compounds like tocopherol homologues, fatty acids, protein, and oil were mapped in a ‘Vivot’ × ‘Blanquerna’ almond population (Font i Forcada et al., 2012; Fernández i Martí et al., 2013). Additionally, association analyses using a wide almond germplasm collection investigated the genetic control of kernel dimensions (width, thickness, and length) and other chemical traits (Font i Forcada et al., 2015a and 2015b). In recent years, new genomic tools have been developed, and three different almond genomes have been released (Sánchez-Pérez et al., 2019; Alioto et al., 2020; D’Amico-Willman et al., 2022; Castanera et al., 2024) along with a 60K SNP array for high-throughput genotyping (Duval et al., 2023). These advances have enabled the development of highly saturated linkage maps used to search for genetic associations with traits like shell hardness (Goonetilleke et al., 2018), the volatilome of roasted kernels (Di Guardo et al., 2021), and other phenological and nut quality traits (Paizila et al., 2021; Goonetilleke et al., 2023; Sideli et al., 2023; Pérez de los Cobos et al., 2023; Sideli et al., 2024). As a result of these efforts, four major genes: self-compatibility, late-blooming, sweet kernel, and shell hardness are currently being selected with molecular markers in different breeding programs (Goonetilleke et al., 2018; Sideli et al., 2023). However, despite the development of a marker for kernel bitterness (Sánchez-Pérez et al., 2010) along with KASP markers (Ricciardi et al., 2018; Lotti et al., 2023), no markers of breeding interest are available for other physicochemical almond kernel traits.

In this study, the genetic inheritance of kernel quality traits such as kernel weight, shape-related traits, color, and chemical composition was investigated in an F_1_ population from the ‘Marcona’ × ‘Marinada’ cross. This population was phenotyped for four years for physical traits and one year for kernel chemical composition. Highly saturated linkage maps were developed using the genotypes obtained with the almond 60K SNP array (Duval et al., 2023) and QTL mapping was carried out.

## 2 Materials and methods

### 2.1 Plant material

An F_1_ population of 91 individuals derived from the cross between ‘Marcona’ and ‘Marinada’ (MC × MI) was used in this study. ‘Marcona’ is a highly valued traditional Spanish cultivar, known for its characteristic rounded kernel shape and high level of fatty acids (Martín Carratalá et al., 1998; Calle et al., 2024). ‘Marinada’ (‘Lauranne’ × ‘Glorieta’) is a self-compatible breeding cultivar released by IRTA in 2008, noted for its very sweet kernel and high percentage of soluble sugars (Vargas et al., 2008; Romero et al., 2011). Seedlings were grafted onto ‘Garnem^®^’ rootstock and planted at 4 × 1.8 m in 2015. The MC × MI population and its parents are maintained at the IRTA Mas Bové experimental station (41.170723 N, 1.172942 E) under standard agricultural practices.

### 2.2 Phenotypic data collection

The MC × MI population and both parents were evaluated for several physical and chemical traits. The physical traits included kernel weight, crack-out percentage, kernel size (length, width, and thickness), kernel shape (roundness, globosity, shoulder, and symmetry), and tegument and kernel color (L*, a*, b*). The kernel chemical traits assessed were kernel protein, fiber and fat content, and fatty acids profile (myristic, palmitic, palmitoleic, margaric, cis-10-heptadecenoic, stearic, oleic, vaccenic, linoelaidic, linoleic, arachidic, cis-11-eicosenoic, and cis-11,14-eicosadienoic).

**Figure 1:**
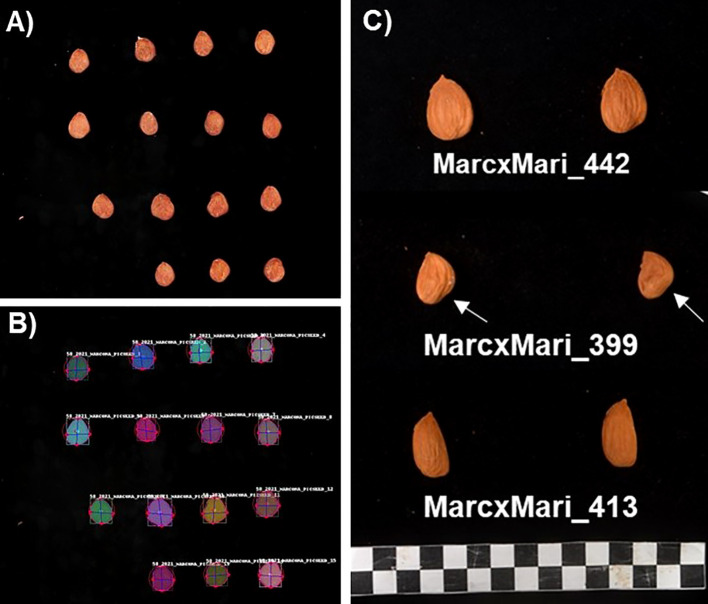
**(A)** Original and **(B)** processed images for ‘Marcona’ shape attributes using the Shape analyzer software. **(C)** Kernel images of three different individuals from the ‘MC × MI’ population where segregation of kernel shape and the shoulder trait (arrow) are shown.

From 2018 to 2021, fifty mature fruits were randomly collected from each individual of the F_1_ MC × MI population and its parents. Fruits were considered mature when the mesocarp was fully dry, split along the fruit suture, and the peduncle was near complete abscission. After dehulling, nut weight was measured, shells were cracked, and kernel weight (KWe) was recorded using an electronic scale. Crack-out percentage, calculated between 2019 and 2021, was determined as the ratio of kernel and nut weights. After that, kernel length (KLen), width (KWidth), and thickness (KThick) was measured with a digital Vernier caliper. Kernel roundness (KRound) and globosity (KGlob) were estimated using the ratios width/length and width/thickness, respectively. Kernel size and shape traits were also assessed using image analysis. For that, a standard photo of six kernels was taken per individual (**Figure 1**), and images were analyzed using the Shape Analyzer (SA) software (Jurado, 2024). This deep learning-based tool automatically detects almond kernels from images and measures them. The parameters obtained included kernel length (KLen_SA), width (KWidth_SA), roundness (KRound_SA), symmetry based on ‘structural similarity index measure’ (SSIM) (Sym_SSIM), and Jaccard index (Sym_Jacc). Both indices determine kernel symmetry by comparing the similarity between the two sides of the kernel along the longitudinal axes using digital images (**Figure 1B**). For that, the box containing the kernel (**Figure 1B**) was divided in half and right-flipped to compare the similarity between both sides using the SSIM and Jaccard coefficient metrics, with a value of 1 indicating perfect symmetry. These traits were phenotyped in 2020 and 2021. Additionally, in 2020, the kernel shoulder, referring to anomalies in the basal area of the kernels (**Figure 1**), was visually assessed using a scale from one (no shoulder) to five (marked basal anomaly as observed in **Figure 1C**; KShoul_Vis), and measured using the Tomato analyzer software (Gonzalo et al., 2009) to determine the angle of the kernel shoulder (KShoul_Angle) as shown in **Figure 1**. Tegument (the kernel skin) and kernel color were determined with a Minolta Chroma Meter tri-stimulus color analyzer (CR-3500D; Minolta, Ramsey, NJ, USA) calibrated to a white porcelain reference plate using a CIELAB scale with color space coordinates L*, a* and b* (Tcolor_L, Tcolor_a, Tcolor_b, Kcolor_L, Kcolor_a, Kcolor_b). Tegument and kernel color were measured from 2018 to 2021.

Fat content was analyzed by Soxhlet method, using 5 – 6 g of ground blanched almonds and petroleum ether (boiling point 40 to 60 °C) for 7 h in Soxhlet apparatus (Romero et al., 2021; Etheridge et al., 1998). The fat content was expressed as a percentage (%), calculated based on the weight of the extracted fat relative to the total weight of the sample. This trait was measured only in 2021.

Crude protein was analyzed from 2018 to 2021 by Dumas combustion procedure using Leco FP-528 analyzer as described in Romero et al. (2021). Briefly, 0.2 g of grounded sample was weighed in a porcelain sample holder (boat) for introduction into the combustion chamber (850 ± 1 °C) utilizing an automated sample loader. The combustion process converts covalently bound 130 nitrogen into nitrogen gas (N_2_) that is quantified by passing the gas through a conductivity cell and converted to protein by multiplying a factor of 6.25. The results were expressed as a percentage (%) of protein content.

Crude fiber was measured from 2019 to 2021 using 1 g of the grounded sample. The sample was treated with boiling 0.26 N sulfuric acid for 30 min, followed by boiling 0.23 N potassium hydroxide for another 30 min (Romero et al., 2021). The extracted residue was dried at 103 ± 1 °C for 3 h, weighed, then placed in a furnace (550 ± 1 °C for 3 h), and finally, the ashes were weighed. These results were expressed as a percentage (%) of ash content.

Fatty acids were analyzed by gas-chromatography with a flame ionization detector (GC-FID) using a capillary column (Romero et al., 2021). The fatty acid methyl esters (FAMEs) were prepared by trans-esterification with 0.5 M potassium hydroxide, following the official method UNE-EN ISO 5509:2000. FAMEs (1 mL) were separated using a gas-chromatograph (HP 6890; Agilent Technologies, Barcelona, Spain) equipped with an FID detector and a capillary column [30 m · 0.25 mm i.d. (HP-Innowax, Agilent Technologies)]. The carrier gas was helium, with a flow rate of 1 mL/min. The injector and detector temperatures were 220 and 275°C, respectively. The FAME identification was based on retention time relative to those of a standard FAME mixture (Sigma-Aldrich, Madrid, Spain). Fatty acids were measured only in 2021. Results were expressed in mg/g as average values of three replicates.

### 2.3 Data analyses

In this study, LSmean, which provides a way to obtain mean trait values that are adjusted for fixed effects, was considered to predict the effect of the year, assuming that the environmental conditions of every year affect all individuals in the population in the same way. Therefore, for traits with more than one year of data, the LSmean was calculated according to the following equation:


$$P_{ij}=\;\beta_{0}+\beta_{i}+\beta_{yearj}+e_{ij}$$

where $P_{ij}$ is the phenotypic value of the i-th individual in the j-th year, $\beta_{0}$ is the intercept (overall mean phenotypic value), $\beta_{i}\;$ is the effect of the i-th individual, $\beta_{yearj}$ is the effect of the j-th year, and $e_{ij}$ is the error term for the i-th individual in the j-th year. The coefficient of determination (R^2^) was used to measure how the LSmean fits the data and how well it can predict outcomes. R^2^ was determined using the following formula:


$$R^{2}=1-SSR–SST$$

where SST is the sum of squares of the residual errors, and SST is the total sum of the errors. R^2^ ranges between 0 and 1, with values closer to 1 indicating better LSmean prediction.

Pairwise correlation coefficients were calculated for all traits (single-year data and LSmean data) using the JMP^®^ software (JMP^®^, Version 16, SAS Institute Inc.) Significance was calculated using the Spearman correlation coefficient significant (p<0.001). Normal distribution was assessed using the Shapiro-Wilk test (p<0.05) using JMP^®^.

### 2.4 Genotyping, SNP filtering, and linkage map construction

Total genomic DNA from the 91 individuals and two parents was isolated from young leaves using the CTAB method (Doyle and Doyle, 1990), adapted for 96-well plates. The DNA samples were genotyped with 33 SSRs and the almond Axiom™ 60K SNP array (Duval et al., 2023).

For the SSRs, a set of markers that were heterozygous in ‘Marcona’ or in ‘Marinada’ was selected. In genomic regions not well covered with the initial set of SSRs, new SSRs were designed using the almond reference genome (Alioto et al., 2020). Primers were designed using Primer 3 (http://primer3.ut.ee, v4.1.0) with default parameters. The list of SSR markers used is presented in **Supplementary Table 1**. PCR reactions were conducted in a final volume of 10 μL containing 200 ng of genomic DNA, 1x NH_4_ reaction buffer, 1.5 mM MgCl_2_, 0.2 mM dNTPs (10mM), 0.2 μM of each marker, 1 U of BIOTaq (Bioline, London, UK) and HPLC H_2_O to reach the final volume. PCRs were performed in a GeneAmp PCR System 9700 thermal cycler (Applied Biosystems, CA, USA) with the following conditions: initial denaturation at 94°C for 1 min, 35 cycles of denaturation at 94°C for 15 s, primer annealing at a specific temperature for each primer for 15 s, extension at 72°C for 30 s, and a final extension at 72°C for 5 min. Forward primers were designed with a generic fluorochrome sequence at the 5’ ends (FAM, VIC, NED, or PET). PCR products were added to 12 μL of deionized formamide containing 0.35 μL of GeneScan500 LIZ size standard (Applied Biosystems, CA, USA) and heated at 94°C for 3 min. Capillary electrophoresis was performed in an ABI Prism 3130xl automated sequencer (Applied Biosystems, CA, USA). GeneMapper v5.0 software (Applied Biosystems) was used for SSR allele sizing.

The almond Axiom™ 60K SNP array genotyping was performed on an Axiom GeneTitan™ (ThermoFisher Scientific) system at the INRAE Gentyane platform in Clermont-Ferrand (France). Genotypic data was retrieved using the Axiom Analysis Suite (ThermoFisher, 2017). Samples were filtered following the Axiom best practices workflow, setting an average call rate > 95. We then filtered out SNPs with the following characteristics: (i) monomorphic SNPs in the progeny; (ii) heterozygous SNPs in ‘Marcona’ and ‘Marinada’ but with only two genotypic classes in the progeny; (iii) homozygous SNPs in ‘Marcona’ and heterozygous in ‘Marinada’ with three genotypic classes in the progeny; (iv) homozygous SNPs in ‘Marinada’ and heterozygous in ‘Marcona’, but with three genotypic classes in the progeny. After filtering based on segregation, missing data, and putative genotyping error identification and imputation were performed with AlphaFamImputed software (Whalen et al., 2020) using default settings. We then ordered the SNPs based on their physical position in the ‘Texas’ almond genome v2.0 (Alioto et al., 2020) and phased them manually. Finally, a set of bins (i.e. groups of SNPs with identical genotypes for all the individuals) was established, with each bin separated from the adjacent bin by at least one recombination event.

Finally, three linkage maps were built using JoinMap 5^®^ (van Ooijen, 2018). The MC × MI map was constructed using all the previously selected bins, the ‘Marcona’ map was built using only bins heterozygous in ‘Marcona’, and the ‘Marinada’ map was built using only bins heterozygous in ‘Marinada’. For map construction, a minimum logarithm of odds (LOD) score of 10 was selected for SNP grouping. Makers showing segregation distortion higher than 0.01 were excluded from linkage mapping unless other distorted markers with similar ratios surrounded them. The maximum likelihood algorithm with default parameters and the Kosambi mapping function were used (Kosambi, 1944).

### 2.5 QTL mapping

LSmean and single-year data were analyzed for QTL detection in the three linkage maps (‘Marcona’, ‘Marinada’, and MC × MI) using MapQTL 6.0^®^ (van Ooijen, 2009). Interval mapping (Lander and Botstein, 1989) and multiple QTL mapping (Jansen and Stam, 1994) strategies were used for QTL discovery. To determine the significance threshold for each QTL, LOD was calculated for each linkage group (LG) and trait using the permutation test (1000 permutations) at a 95% significance level (p < 0.05) (Lander and Botstein, 1989; van Ooijen, 1992). As most traits had a significance level similar to 3, a final threshold of LOD 3 was used in all cases. The QTL confidence interval was defined as the LOD - 1. For traits with non-normally distributed data, we used the Kruskal-Wallis non-parametric test in MapQTL, retaining only QTLs that were significant in this test (p< 0.01) and also significant with interval mapping (LOD > 3.0). QTLs were named according to the recommendations for standard QTL nomenclature and reporting of the Genome Database for Rosaceae (Jung et al., 2019). Graphical representation of linkage maps and QTLs was created using MapChart (Voorrips, 2002). The physical positions of the QTLs are based on the almond reference genome of ‘Texas’ v3.0-F1 (Castanera et al., 2024).

## 3 Results

### 3.1 Trait distributions and correlations

We obtained phenotypic data for 22 traits, including both kernel physical and chemical characteristics. The physical traits measured were kernel weight, crack-out percentage (crack-out), kernel size (length, width, and thickness), kernel shape (roundness, globosity, shoulder, and symmetry), and tegument and kernel color (L*, a*, b*). The kernel chemical traits included kernel protein, fiber, and fat content. For the fatty acid profile, we measured myristic, palmitic, palmitoleic, margaric, cis-10-heptadecenoic, stearic, oleic, vaccenic, linoelaidic, linoleic, arachidic, cis-11-eicosenoic, and cis-11,14-eicosadienoic acids.

#### 3.1.1 Trait distributions

**Figure 2:**
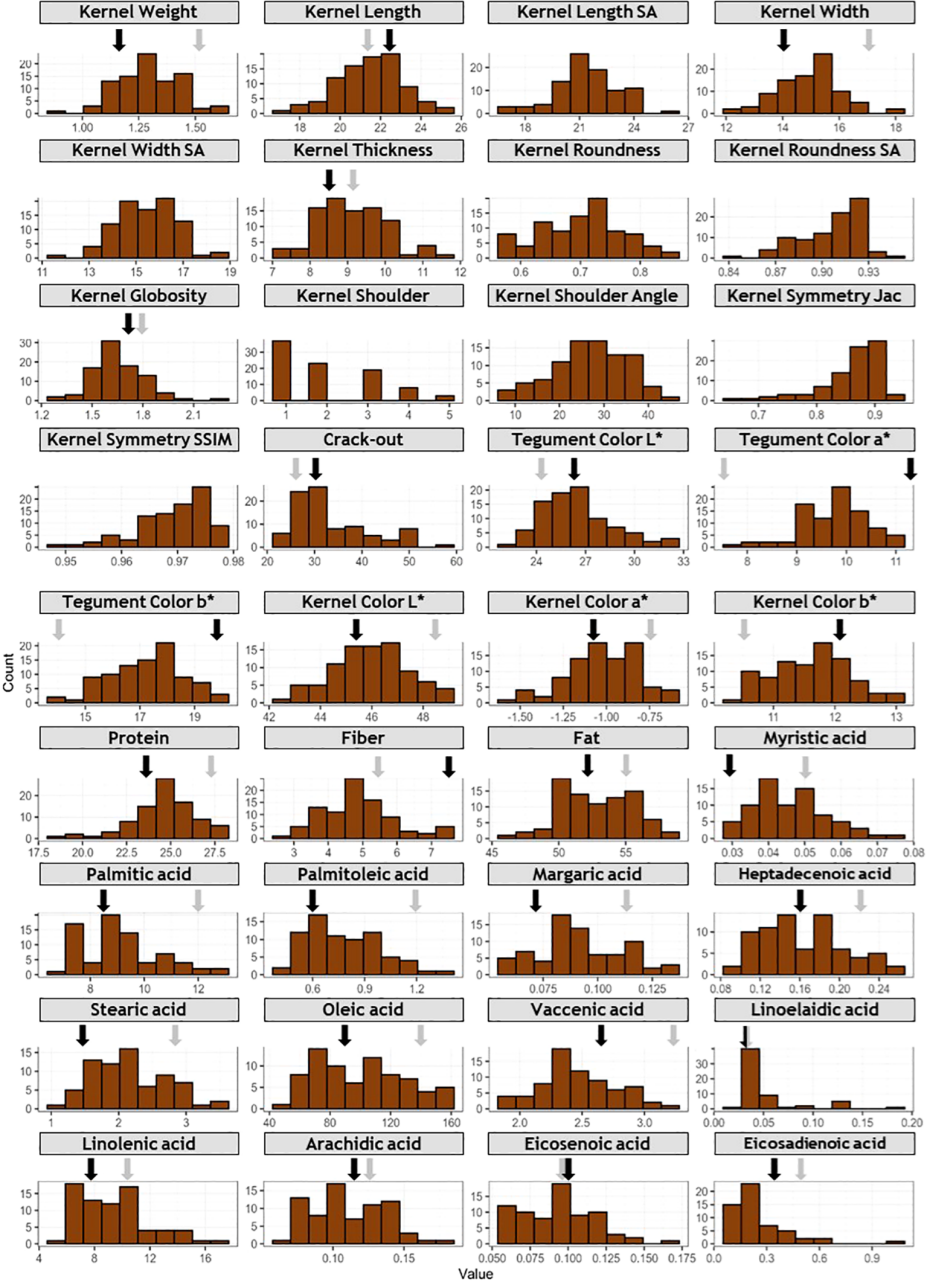
Frequency distribution of phenotyped traits. For traits with more than one year of data, LSmean values are used. *X* axis represents phenotypic values, and *Y* axis represents frequency. Grey and black arrows indicate phenotypic values for ‘Marcona’ and ‘Marinada’, respectively.

‘Marcona’ and ‘Marinada’ showed intermediate values for most traits, however ‘Marcona’ displayed high kernel width and protein content compared to the population mean (**Figure 2**; **Supplementary Table 2**). In general, ‘Marcona’ had a larger kernel weight, a rounder kernel (wider but with a similar length), a lower crack-out percentage, and a higher protein content compared to ‘Marinada’.

Concerning fruit dimensions, individuals of the ‘Marcona’ × ‘Marinada’ (MC × MI) population exhibited weights ranging from 0.89 to 1.61 g, lengths from 16.77 to 24.87 mm, widths from 12.17 to 17.95 mm, and thicknesses from 7.21 to 11.56 mm (**Figure 2**; **Supplementary Table 2**). In terms of roundness (width/length ratio) and globosity (width/thickness ratio), the trait distributions spanned from 0.56 to 0.84 mm and 1.31 to 2.27 mm, respectively, with a larger number of individuals showing elongated shapes compared to rounded shapes (**Figure 2**). Similarly, there was an evident segregation for the shoulder trait, with a higher frequency of fruits with less pronounced shoulders. For symmetry, measured using Jaccard and SSIM indexes, a clear trend towards fruits with high symmetry was observed. For crack-out percentage, a distribution biased towards fruits with hard shell (<40%) was observed.

Regarding the chemical composition of the kernel, protein content varied between 18.47 and 27.72%, fiber content ranged from 2.80 to 7.56%, and fat content ranged from 46.62 to 58.83% (**Figure 2**). In the individuals of the population, a distribution towards higher levels of protein and fat content was observed. However, the distribution of fiber content exhibited a slightly different pattern towards lower amounts. The predominant fatty acid detected was oleic (ranging from 52.90 to 160.85 mg/g), followed by linolenic (5.33-17.12 mg/g), palmitic (6.78-12.84 mg/g), stearic (1.03-3.45 mg/g), and vaccenic (1.83-3.15 mg/g). Other identified acids, such as myristic, palmiloleic, margaric, heptadeceonic, linoelaidic, arachidic, eicosenoic, and eicosadienoic, presented concentrations lower than 1.5 mg/g (**Figure 2**).

Regarding the color of the tegument, a distribution towards nuts with darker pigmentation was noted. In contrast, for nuts without the tegument, minimal differences were observed among the samples.

The normality of each trait was evaluated using the Shapiro-Wilk test (**Supplementary Table 2**). Among the kernel dimension traits, all followed a normal distribution except for roundness, symmetry, and kernel shoulder (p< 0.01). Kernel weight also displayed a normal distribution (W = 0.987, p = 0.514). For tegument and kernel color traits, only Tegument Color L* significantly deviated from normality. In terms of chemical composition, fat content followed a normal distribution, in contrast to protein and fiber content (**Supplementary Table 2**). Among the fatty acids, only palmitoleic, stearic, and vaccenic acids exhibited a normal distribution pattern.

#### 3.1.2 Trait correlations

The coefficient of determination (R^2^) was calculated for all traits phenotyped over multiple years to assess the predictive accuracy of LSmean values (**Supplementary Table 3**). Overall, a high prediction was observed for all traits. The lowest prediction values were observed for fiber content (R^2^ = 0.45) and tegument color L* (0.47) (**Supplementary Table 3**). Conversely, all the other traits presented R^2^ values higher than 0.64 with the highest values recorded for roundness-SA and symmetry-Jaccard-SA (0.91).

Significant correlations between years were obtained for all traits, except for tegument and kernel color parameters, which were only significant between 2020 and 2021 (**Supplementary Table 4**). Moreover, for all the other traits, higher correlations were observed between 2020 and 2021 compared to those between 2018 and 2019. The highest inter-annual correlations were observed for crack-out percentage (0.89) and kernel roundness (0.88) between 2020 and 2021, while the lowest correlation was for kernel thickness (0.25) between 2018 and 2019.

Several sets of correlated traits were identified in the population (**Supplementary Table 4**). One set focused on traits associated with kernel dimensions. Considering only comparisons between LSmeans of all traits, kernel weight demonstrated intermediate to high correlations with both kernel length and width, using measurements from both manual assessments (0.52 and 0.60, respectively) and outputs from the Shape Analyzer (SA) software (0.62 and 0.67, respectively). Conversely, a low correlation of 0.27 was reported between kernel weight and thickness. Regarding kernel length, a moderate positive correlation with weight (0.52) was observed, along with a negative correlation with roundness (-0.67). Interestingly, no significant correlation between kernel length and width was noted, in contrast to the positive correlation between width and roundness (0.60) and roundness-SA (0.52). Kernel width also exhibited a moderate correlation with thickness (0.48), which was correlated with roundness (0.48) and kernel shoulder (0.53 and 0.46 for angle and visual, respectively). As expected, high correlations were observed for kernel length (0.82; KLength vs. KLenght_SA), width (0.83; KWidth vs. KWidth_SA), and roundness (0.67; KRound vs. KRound_SA) when comparing manual measures to outputs from the Shape Analyzer software (**Supplementary Table 4**).

A different set of interrelated traits was documented for chemical compounds (**Supplementary Table 4**). Notably, a negative correlation was identified between fat and protein content (-0.61), contrasting with the consistently high positive correlations among all analyzed fatty acids (ranging from 0.33 to 0.92). There was a low and non-significant correlation observed between fat, fiber, and protein content, and the fatty acids (**Supplementary Table 4**). Additionally, negative correlations between certain chemical and physical parameters were noted, such as crack-out percentage and protein content (-0.34) and fat content and globosity (-0.47).

High correlations were also observed between color parameters like tegument color a* and tegument color L* (-0.44), and tegument color a* and tegument color b* (0.55) (**Supplementary Table 4**).

### 3.2 Genotyping and linkage map construction

A total of 9243 SNPs, representing 15.3% of the almond Axiom™ 60K SNP array, were obtained from the SNP filtering procedures and used for linkage mapping along with 33 simple-sequence repeat (SSR) markers. Of these SNPs, 3810 were heterozygous in ‘Marcona’ and homozygous in ‘Marinada’, 4060 were heterozygous in ‘Marinada’ and homozygous in ‘Marcona’, and the remaining 1373 SNPs were heterozygous in both parents. Additionally, 33 SSR markers were included.

For all constructed linkage maps (‘Marcona’, ‘Marinada’, and ‘MC × MI’), markers were grouped into eight LGs, each corresponding to an almond chromosome (**Supplementary Table 5**). The ‘Marcona’ map covered 448.0 cM and presented 370 bins (i.e., groups of markers in unique genetic positions) (**Table 1**). Similarly, the ‘Marinada’ map had 316 bins distributed along 447.0 cM. The ‘MC × MI’ showed higher marker saturation than parental maps, with 685 bins covering 540.8 cM (**Table 1**). In the three linkage maps, LG1 covered the largest genetic distance and included the highest number of markers. The average distance between consecutive bins was less than 1 cM in the ‘MC × MI’ map, whereas for the parental maps, this distance ranged from 1.15 (‘Marcona’; LG1) to 1.61 cM (‘Marinada’; LG7) (**Table 1**). Additionally, the linkage maps showed good genome coverage without major gaps: the average physical distance between consecutive markers was 196, 555, and 612 Kbp in the ‘MC × MI’, ‘Marcona’, and ‘Marinada’ maps, respectively.

**Table 1:** Summary statistics for the ‘Marcona’, ‘Marinada’ and ‘Marcona’ × ‘Marinada’ (MC × MI) genetic maps.

	Linkage map	LG1	LG2	LG3	LG4	LG5	LG6	LG7	LG8	Total
Number of bins	Marcona	64	49	45	51	35	35	50	41	370
	Marinada	64	39	38	37	32	37	33	36	316
	MC × MI	129	88	79	89	70	72	84	74	685
Genetic distance (cM)	Marcona	72.4	63.6	58.2	71.7	51.9	44.5	62.5	53.2	448.0
	Marinada	91.8	55.3	52.8	51.3	40.8	51.6	51.7	51.7	447.0
	MC × MI	84.9	59.4	71.7	73.2	70.8	55.3	64.9	60.6	540.8
Average distance between loci (cM)	Marcona	1.15	1.33	1.32	1.43	1.53	1.31	1.28	1.33	1.34
	Marinada	1.46	1.45	1.43	1.42	1.31	1.43	1.61	1.50	1.45
	MC × MI	0.66	0.68	0.91	0.83	1.02	0.78	0.78	0.83	0.81
Max. gap between markers (cM)	Marcona	2.3	5.5	3.2	3.6	4.7	2.4	2.4	2.4	3.31
	Marinada	5.6	2.4	2.4	3.6	3.0	4.7	4.8	3.6	3.76
	MC × MI	1.7	2.6	4.7	2.9	4.2	2.9	4.9	2.3	3.28

These maps were constructed with one genotype per bin of the 1:1 markers segregating in each of the parents and those plus the 1:2:1 markers identifying new bins in the ‘MC × MI’ map.

### 3.3 QTL mapping

QTL analyses were performed using single-year data for all traits, and for those traits with more than one year of data, LSmean values were also used. All QTLs are presented in **Supplementary Table 6**. In most cases, LSmean data identified the most consistent QTLs, which were detected in at least two different years through single-year QTL mapping. To simplify data presentation, we present QTLs using LSmean and those for which only one year of phenotypic data was available (**Table 2**).

**Table 2:** QTL mapping results using single year phenotypic data or, for traits phenotyped for more than one year, least square (LS) estimates.

Trait	QTL name	Map	Phenotypic data	Max LOD	LG	Closest marker	Physical position (Texas v3.0-F1)	Interval (cM; LOD+/-1)	R^2^	Additive
Kernel Weight										
Kernel Weight	*qP-KWe3.1*	Marcona	LSmean	3.2	3	AX-586061740	Chr03_1849248	0.0-11.4	15.4	0.11
Kernel Weight	*qP-KWe4.1*	Marinada	LSmean	5.7	4	AX-586079155	Chr04_2621791	0.0-6.4	26.1	0.15
Kernel Weight	*qP-KWe4.1*	MC×MI	LSmean	6.5	4	AX-586078061	Chr04_2548827	0.0-9.7	29.0	–
Crackout										
Crackout	*qP-Crack2.1*	Marcona	LSmean	5.1	2	AX-586057484	Chr02_28853706	47.4-60.1	23.4	-7.31
Crackout	*qP-Crack2.1*	Marinada	LSmean	3.5	2	AX-586052317	Chr02_23294133	37.8-49.3	16.7	-6.20
Crackout	*qP-Crack2.1*	MC×MI	LSmean	17.2	2	AX-586052397	Chr02_23428499	42.3-46.3	59.7	–
Kernel size and shape										
Kernel Lenght	*qP-KLen1.1*	Marinada	LSmean	5.6	1	AX-586017842	Chr01_8263778	10.4-19.5	25.8	-1.41
Kernel Length	*qP-KLen1.1*	MC×MI	LSmean	7.3	1	AX-586018172	Chr01_9436655	11.4-19.2	32.1	–
Kernel Width	*qP-KWidth1.1*	Marcona	LSmean	3.8	1	AX-586030939	Chr01_35727471	42.4-50.5	18.3	0.95
Kernel Width	*qP-KWidth8.1*	Marcona	LSmean	3.7	8	AX-586146402	Chr08_15661703	11.6-20.9	17.8	-0.94
Kernel Width	*qP-KWidth4.1*	Marinada	LSmean	3.3	4	AX-586077840	Chr04_2108924	0.0-5.4	15.8	0.89
Kernel Width	*qP-KWidth1.1*	MC×MI	LSmean	4.9	1	AX-586030002	Chr01_34764133	45.3-57.4	22.9	–
Kernel Width	*qP-KWidth7.1*	MC×MI	LSmean	3.7	7	AX-586138643	Chr07_22970625	46.6-65.0	17.9	–
Kernel Width	*qP-KWidth8.1*	MC×MI	LSmean	3.9	8	AX-586146402	Chr08_15661703	17.5-25.0	18.7	–
Kernel Thickness	*qP-KThick1.1*	Marcona	LSmean	4.4	1	AX-586020091	Chr01_12777516	17.5-28.0	20.6	0.78
Kernel Thickness	*qP-KThick4.1*	Marinada	LSmean	4.2	4	AX-586079155	Chr04_2621791	0.0-6.4	20.0	0.82
Kernel Thickness	*qP-KThick1.1*	MC×MI	LSmean	5.5	1	AX-586021692	Chr01_19228013	21.8-29.6	25.2	–
Kernel Thickness	*qP-KThick4.1*	MC×MI	LSmean	5.3	4	AX-586083366	Chr04_8841873	25.3-35.4	24.6	–
Kernel Roundness	*qP-KRound1.1*	Marcona	LSmean	4.5	1	AX-586018708	Chr01_10422924	5.8-24.4	21.0	0.06
Kernel Roundness	*qP-KRound7.1*	Marcona	LSmean	4.8	7	AX-586136730	Chr07_21376413	42.6-55.3	22.3	-0.06
Kernel Roundness	*qP-KRound1.1*	Marinada	LSmean	4.1	1	AX-586016559	Chr01_6865271	5.5-17.1	19.6	0.06
Kernel Roundness	*qP-KRound1.1*	MC×MI	LSmean	7.8	1	AX-586016378	Chr01_6661966	6.3-19.7	33.9	–
Kernel Roundness	*qP-KRound6.1*	MC×MI	LSmean	4.2	6	AX-586109783	Chr06_6435616	3.3-14.8	20.1	–
Kernel Roundness	*qP-KRound7.1*	MC×MI	LSmean	7.2	7	AX-586135231	Chr07_22199172	49.1-54.9	27.5	–
Kernel Globosity	*qP-KGlob4.1*	Marcona	LSmean	4.0	4	AX-586087837	Chr04_15732422	48.4-62.5	19.4	0.14
Kernel Globosity	*qP-KGlob2.1*	MC×MI	LSmean	5.0	2	AX-586052397	Chr02_23428499	40.0-52.6	23.3	–
Kernel Globosity	*qP-KGlob3.1*	MC×MI	LSmean	3.8	3	AX-586074007	Chr03_26939315	57.2-71.5	18.1	–
Kernel Globosity	*qP-KGlob4.1*	MC×MI	LSmean	4.6	4	CPPCT046	Chr04_16391606	38.9-65.0	21.5	–
Kernel Lenght_SA	*qP-Klen_SA1.1*	Marinada	LSmean	6.3	1	AX-586016345	Chr01_6488397	1.2-10.4	28.5	-1.53
Kernel Lenght_SA	*qP-Klen_SA1.1*	MC×MI	LSmean	6.8	1	AX-586016345	Chr01_6488397	3.4-9.1	31.2	–
Kernel Lenght_SA	*qP-Klen_SA6.1*	MC×MI	LSmean	4.2	6	AX-586106625	Chr06_1988726	3.3-16.8	20.2	–
Kernel Width_SA	*qP-Kwidth_SA8.1*	Marcona	LSmean	4.3	8	AX-586146402	Chr08_15661703	10.5-24.4	20.5	-1.13
Kernel Width_SA	*qP-Kwidth_SA1.1*	MC×MI	LSmean	4.4	1	AX-586030002	Chr01_34764133	47.8-56.4	20.8	–
Kernel Width_SA	*qP-Kwidth_SA2.1*	MC×MI	LSmean	3.8	2	AX-586053857	Chr02_25390538	47.4-52.6	18.3	–
Kernel Width_SA	*qP-Kwidth_SA4.1*	MC×MI	LSmean	4.7	4	AX-159226595	Chr04_1197932	3.3-9.7	20.0	–
Kernel Width_SA	*qP-Kwidth_SA7.1*	MC×MI	LSmean	3.8	7	AX-586134161	Chr07_21457125	46.6-64.9	18.5	–
Kernel Width_SA	*qP-Kwidth_SA8.1*	MC×MI	LSmean	4.7	8	AX-586145789	Chr08_14603868	14.6-30.2	22.0	–
Kernel Roundness_SA	*qP-Kround_SA7.1*	Marcona	LSmean	4.3	7	AX-586136730	Chr07_21376413	42.6-54.2	20.7	-0.02
Kernel Roundness_SA	*qP-Kround_SA2.1*	Marinada	LSmean	3.8	2	AX-586047267	Chr02_15891435	10.5-26.9	18.3	0.02
Kernel Roundness_SA	*qP-Kround_SA2.1*	MC×MI	LSmean	4.0	2	AX-586046794	Chr02_15813584	10.8-25.0	19.3	–
Kernel Roundness_SA	*qP-Kround_SA6.1*	MC×MI	LSmean	4.4	6	AX-586105710	Chr06_3532533	3.3-13.0	21.1	–
Kernel Roundness_SA	*qP-Kround_SA7.1*	MC×MI	LSmean	5.1	7	AX-586137299	Chr07_21702235	46.9-54.3	23.8	–
Sym_SSIM_SA	*qP-Sym_SSIM1.1*	Marcona	LSmean	3.1	1	AX-586025379	Chr01_27873984	36.5-45.8	15.2	-0.01
Sym_SSIM_SA	*qP-Sym_SSIM1.1*	Marinada	LSmean	4.6	1	AX-586025148	Chr01_27294920	34.8-42.4	21.9	-0.01
Sym_SSIM_SA	*qP-Sym_SSIM1.1*	MC×MI	LSmean	9.4	1	AX-586025038	Chr01_27047791*	38.7-40.9	39.5	–
Sym_SSIM_SA	*qP-Sym_SSIM8.1*	MC×MI	LSmean	4.2	8	AX-586139063	Chr08_1942794	4.1-12.6	20.3	–
Sym_Jaccard_SA	*qP-Sym_Jacc1.1*	Marcona	LSmean	3.2	1	AX-586025379	Chr01_27873984	36.5-47.0	15.9	-0.04
Sym_Jaccard_SA	*qP-Sym_Jacc1.1*	Marinada	LSmean	4.9	1	AX-586025148	Chr01_27294920	23.5-42.4	23.1	-0.05
Sym_Jaccard_SA	*qP-Sym_Jacc1.1*	MC×MI	LSmean	9.4	1	AX-586025038	Chr01_27047791*	38.7-40.9	39.6	–
Sym_Jaccard_SA	*qP-Sym_Jacc8.1*	MC×MI	LSmean	4.4	8	AX-586141494	Chr08_1101159	5.2-10.3	22.7	–
Kernel Shoulder	*qP-KShoul_Vis1.1*	Marcona	2020	4.2	1	AX-586026674	Chr01_29944777	36.5-49.3	19.9	1.00
Kernel Shoulder	*qP-KShoul_Vis8.1*	Marcona	2020	3.3	8	AX-586139190	Chr08_2202923	0.0-8.0	15.8	-0.90
Kernel Shoulder	*qP-KShoul_Vis1.1*	Marinada	2020	7.9	1	AX-586025148	Chr01_27294920	37.8-42.4	34.1	1.14
Kernel Shoulder	*qP-KShoul_Vis1.1*	MC×MI	2020	11.5	1	AX-586025171	Chr01_27336819	38.7-40.9	45.7	–
Kernel Shoulder	*qP-KShoul_Vis8.1*	MC×MI	2020	4.1	8	AX-586139429	Chr08_3696522	4.1-11.3	19.5	–
Kernel Shoulder_angle	*qP-KShoul_Angle1.1*	Marcona	2020	4.7	1	AX-586025379	Chr01_27873984	34.1-45.6	22.3	7.74
Kernel Shoulder_angle	*qP-KShoul_Angle1.1*	Marinada	2020	6.4	1	AX-586016559	Chr01_6865271	7.0-18.5	29.4	8.80
Kernel Shoulder_angle	*qP-KShoul_Angle1.1*	MC×MI	2020	10.7	1	CPPCT027	Chr01_14035255	24.5-26.8	43.9	–
Kernel Colour										
Kernel_a	*qP-Kcolor_a1.1*	Marinada	LSmean	4.8	1	AX-586015732	Chr01_5495727	0.0-3.5	22.3	-0.19
Kernel_a	*qP-Kcolor_a1.1*	MC×MI	LSmean	5.5	1	AX-586014695	Chr01_4179608	1.1-6.4	25.3	–
Kernel_b	*qP-Kcolor_b2.1*	Marinada	LSmean	5.3	2	AX-586049906	Chr02_21476757*	30.7-38.8	24.5	0.60
Kernel_b	*qP-Kcolor_b2.1*	MC×MI	LSmean	6.0	2	AX-586057486	Chr02_28856443	44.0-57.0	27.1	–
Kernel composition										
Protein	*qP-Protein2.1*	MC×MI	LSmean	4.5	2	AX-586053334	Chr02_24572305	42.3-50.9	21.2	–
Protein	*qP-Protein3.1*	MC×MI	LSmean	3.8	3	AX-586075035	Chr03_27000444	56.1-71.7	18.4	–
Protein	*qP-Protein5.1*	MC×MI	LSmean	3.7	5	AX-586102987	Chr05_18821223	61.5-69.4	17.6	–
Protein	*qP-Protein7.1*	MC×MI	LSmean	3.5	7	AX-586132921	Chr07_17547713	28.5-37.5	16.9	–
Fiber	*qP-Fiber1.1*	MC×MI	LSmean	3.4	1	AX-586018168	Chr01_9432165	14.8-23.3	16.2	–
Fat	*qP-Fat3.1*	MC×MI	2021	3.9	3	AX-586061116	Chr03_366840	0.0-3.7	19.5	–
Fat	*qP-Fat5.1*	MC×MI	2021	3.9	5	AX-586103462	Chr05_19354347	48.1-70.8	19.4	–
Myristic acid	*qP-MyrisitcAc1.1*	Marinada	2021	3.4	1	AX-159179465	Chr01_49846435	81.2-91.8	17.7	-0.01
Myristic acid	*qP-MyrisitcAc1.1*	MC×MI	2021	3.8	1	AX-159179465	Chr01_49846435	74.7-85.0	19.7	–
Palmitic acid	*qP-PalmiticAc1.1*	Marcona	2021	3.5	1	AX-586034706	Chr01_40645292*	49.5-58.7	18.4	1.21
Palmitic acid	*qP-PalmiticAc1.1*	Marinada	2021	3.9	1	AX-586041516	Chr01_49772602	83.7-91.8	20.3	-1.27
Palmitic acid	*qP-PalmiticAc1.1*	MC×MI	2021	6.8	1	AX-586036301	Chr01_43754489	69.6-74.1	32.7	–
Palmitoleic acid	*qP-PalmitoleicAc1.1*	Marinada	2021	3.4	1	AX-586041516	Chr01_49772602	75.4-91.8	17.7	-0.17
Palmitoleic acid	*qP-PalmitoleicAc1.1*	MC×MI	2021	4.7	1	AX-586041439	Chr01_49655891	69.6-85.0	24.0	–
Margaric acid	*qP-MargaricAc1.1*	Marcona	2021	3.4	1	AX-586034706	Chr01_40645292*	50.7-61.1	17.9	0.02
Margaric acid	*qP-MargaricAc1.1*	Marinada	2021	5.6	1	AX-586041516	Chr01_49772602	84.9-91.8	27.6	-0.02
Margaric acid	*qP-MargaricAc1.1*	MC×MI	2021	8.2	1	AX-586036301	Chr01_43754489	67.9-72.6	37.9	–
Heptadecenoic acid	*qP-HeptadeAc1.1*	Marinada	2021	5.5	1	AX-586041516	Chr01_49772602	84.9-91.8	27.2	-0.04
Heptadecenoic acid	*qP-HeptadeAc1.1*	MC×MI	2021	7.1	1	AX-586041439	Chr01_49655891	81.5-85.0	33.8	–
Stearic acid	*qP-StearicAc1.1*	Marinada	2021	6.0	1	AX-586038470	Chr01_46296562	83.7-91.8	29.5	-0.56
Stearic acid	*qP-StearicAc1.1*	MC×MI	2021	6.9	1	AX-586037985	Chr01_45765548	77.6-81.0	33.1	–
Linoleic acid	*qP-LinoleicAc1.1*	Marinada	2021	3.5	1	AX-586041516	Chr01_49772602	83.7-91.8	18.6	-1.98
Linoleic acid	*qP-LinoleicAc1.1*	MC×MI	2021	7.7	1	AX-586036301	Chr01_43754489	69.6-73.4	39.0	–
Oleic acid	*qP-OleicAc1.1*	Marcona	2021	3.0	1	AX-586033374	Chr01_38970432	40.0-58.7	15.9	22.53
Oleic acid	*qP-OleicAc1.1*	Marinada	2021	5.2	1	AX-586041516	Chr01_49772602	83.7-91.8	26.2	-28.7
Oleic acid	*qP-OleicAc1.1*	MC×MI	2021	7.0	1	AX-586041439	Chr01_49655891	81.0-85.0	33.3	–
Vaccenic acid	*qP-VaccenicAc1.1*	MC×MI	2021	4.6	1	AX-586036301	Chr01_43754489	67.8-75.8	26.7	–
Arachidic acid	*qP-ArachidicAc1.1*	Marinada	2021	4.2	1	AX-586041516	Chr01_49772602	83.7-91.8	22.4	-0.02
Arachidic acid	*qP-ArachidicAc1.1*	MC×MI	2021	5.5	1	AX-586036301	Chr01_43754489	69.6-72.6	27.4	–
Eicosenoic acid	*qP-EicoAc1.1*	Marinada	2021	4.1	1	AX-586041516	Chr01_49772602	83.7-91.8	25.3	-0.02
Eicosenoic acid	*qP-EicoAc1.1*	MC×MI	2021	5.2	1	AX-586041439	Chr01_49655891	75.6-85.0	26.2	–

Physical position based on the ‘Texas’ almond genome v3.0-F1 (Castanera et al., 2024).

* Closest physical position in the almond ‘Texas-V3-F1’ genome. LG: Linkage group. R^2^: Percentage of phenotypic variance explained by QTL.

**Figure 3:**
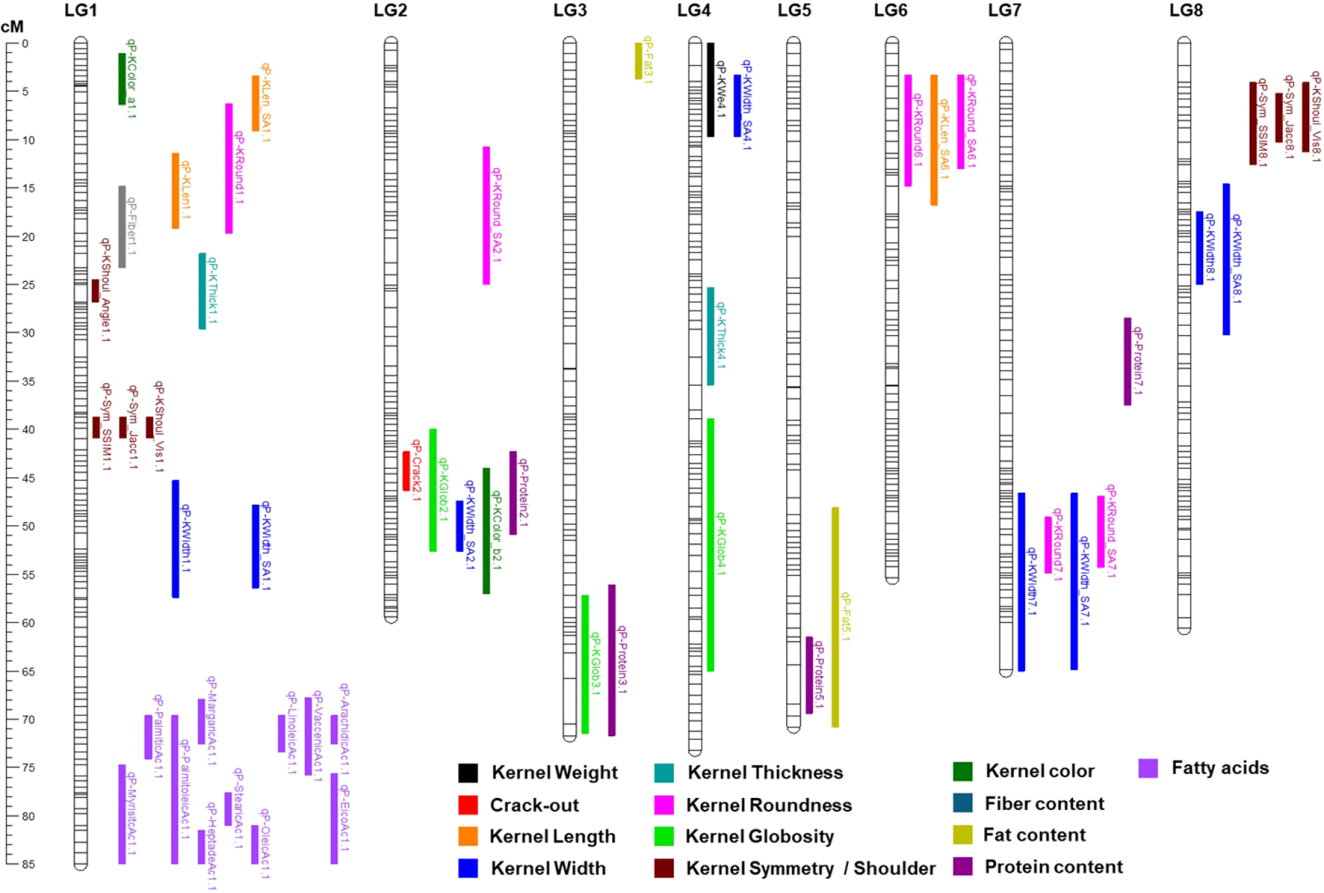
Genetic position (cM) on the ‘Marcona’ × ‘Marinada’ map of detected QTLs using LSmean and those for which only one year of data was available.

In total, 51, 18, and 24 QTLs were detected using the MC × MI, ‘Marcona’, and ‘Marinada’ genetic maps, respectively. After excluding QTLs that cosegregate across different maps, a total of 53 unique QTLs were identified (**Table 2**). Most QTLs detected with the parental maps were also identified with the MC × MI map, except *qP-KWe3.1* (only found on ‘Marcona’ map) and *qP-KWidth4.1* (only found on ‘Marinada’ map). Conversely, 20 QTLs (*qP-KWidth7.1*, *qP-KRound6.1*, *qP-KGlob2.1*, *qP-KGlob3.1*, *qP-KLen_SA6.1*, *qP-KWidth_SA1.1*, *qP-KWidth_SA2.1*, *qP-KWidth_SA4.1*, *qP-KWidth_SA7.1*, *qP-KRound_SA6.1*, *qP-Sym_SSIM8.1*, *qP-Sym_Jacc8.1*, *qP-Protein2.1*, *qP-Protein3.1*, *qP-Protein5.1*, *qP-Protein7.1*, *qP-Fiber1.1*, *qP-Fat3.1*, *qP-Fat5.1*, *qP-VaccenicAc1.1*) were identified on MC × MI map but not on the parental maps. All consistent QTLs identified with the MC × MI map are presented in **Figure 3**.

At least one QTL was identified for all traits except for the color parameters Tegument_L, Tegument_a, Tegument_b, Kernel_L, and linoelaidic acid. For kernel weight, two regions were detected on LGs 3 and 4 (**Table 2**), with the major QTL (*qP-KWe4.1*) found at the beginning of LG4, explaining 29.0% of the phenotypic variation (PV) in the MC × MI map and 26.1% in the ‘Marinada’ map. This QTL showed an additive effect of the ‘Marinada’ allele (+0.11 g) increasing kernel size in the population (**Table 2**). A less significant weight QTL, only detected in the ‘Marcona’ map, was also identified on LG3 (*qP-We3.1*; PV=15.4%). For crack-out percentage, a highly significant QTL (*qP-Crack2.1*) located on LG2 was detected in the three maps with a LOD of 17.2 and explaining 59.7% of the PV in the MC × MI map (**Table 2**; **Figure 3**).

For kernel size and shape traits, a major QTL was detected at the beginning of LG1 (11.4-19.2 cM) for kernel length (*qP-KLen1.1*) using the ‘Marinada’ and the MC × MI maps, explaining up to 32.1% of the PV. Similarly, another major QTL for kernel width (*qP-KWidth1.1*) was identified in the central part of the same LG1 (45.3-57.4 cM) in the ‘Marcona’ (LOD=3.8; PV=18.3%) and MC × MI (LOD=4.9; PV=22.9%) maps. Overlapping with *qP-KLen1.1*, other consistent QTLs were identified for kernel roundness (*qP-KRound1.1*; LOD=4.1 to 7.8; PV=19.6 to 33.9%) and kernel length_SA (*qP-KLen_SA1.1*; LOD=6.3 to 6.8; PV=28.5 to 31.2%). In the same region of *qP-KWidth1.1*, we identified other QTLs for kernel width SA (*qP-KWidth_SA1.1*), kernel symmetry SSIM (*qP-Sym_SSIM1.1*), kernel symmetry Jaccard (*qP-Sym_Jacc1.1*), and kernel shoulder (*qP-KShoul_Vis1.1*) (**Table 2**; **Figure 3**). For kernel thickness and kernel shoulder angle, QTLs (*qP-KThick1.1* and *qP-KShoul_Angle1.1*) were identified in positions near *qP-KLen1.1*; however, the LOD plots do not clearly reveal whether these represent the same QTL or two distinct QTLs located in very close proximity (**Figure 3**). Other stable QTLs for kernel width (LOD = 3.3 to 3.9; PV = 15.8 to 18.7%) were also identified on LGs 4, 7 and 8 (*qP-KWidth4.1*, *qP-KWidth7.1*, and *qP-KWidth8.1*). Overlapping with *qP-KWidth4.1*, a consistent QTL for kernel thickness (*qP-KThick4.1*) was also identified in ‘Marinada’ (LOD=4.2; PV=20.0%) and MC × MI (LOD=5.3; PV=24.6%) maps (**Table 2**; **Figure 3**). Additional regions with kernel globosity QTLs were reported on LGs 2, 3, and 4, each showing similar LOD scores (3.8-5.0) and PV percentages (18.1-23.3%). Comparison of detected QTLs for kernel length, width, and roundness using manual determinations and Shape Analyzer (SA) software measurements revealed QTLs in similar positions, with only a few QTLs detected exclusively by one method or the other (*qP-KRound1.1*, *qP-Round_SA2.1*, *qP-Width_SA2.1*, *qP-KWidth_SA4.1*, *qP-KLen_SA6.1*).

Regarding the kernel chemical composition, four QTLs for protein content were mapped on LGs 2, 3, 5, and 7 on the MC × MI map (**Table 2**; **Figure 3**). Among them, *qP-Protein2.1* showed the highest LOD scores (4.5) and explained the largest proportion of PV (21.2%). The other three additional protein content QTLs (*qP-Protein3.1*, *qP-Protein5.1*, and *qP-Protein7.1*) were detected with lower significance, having LOD scores below 4 and explaining no more than 19% of the phenotypic variance (**Table 2**). For fat content, two QTLs were found on LGs 3 and 5 of the MC × MI map, both with the same significance (LOD=3.9) and explaining similar PV (19%) (**Table 2**). Notably, the protein and fat content QTLs on LG5 (*qP-Protein5.1* and *qP-Fat5.1*) overlapped in the same region (**Figure 3**). For fiber content, a unique QTL mapped on LG1 (14.8-23.3 cM) of the MC × MI map was detected, associated with a LOD score of 3.4 and explained 16.2% of PV.

For the fatty acid content, QTLs were detected for all compounds except linoelaidic acid, on MC × MI map within the same region at the bottom of LG1 (70-85 cM). The QTL with the highest LOD score and PV explained for these acids was reported for margaric acid (LOD=8.2; PV=37.9%), whereas the mystiric acid QTL presented the lowest significance (LOD=3.8; PV=19.7%). On the ‘Marinada’ map, all the same QTLs for fatty acids reported in the MC × MI map were mapped to the same position of LG1. The ‘Marinada’ allele for these QTLs was associated with reductions in the mean values of all acids within the population (**Table 2**). On the ‘Marcona’ map, only QTLs for palmitic acid, margaric acid and oleic acid were detected, all found on the same LG1 interval as for MC × MI and ‘Marinada’ maps, with PV not exceeding the 20% (**Table 2**). For these QTLs, the ‘Marcona’ allele was associated with increasing in fatty acid content. This effect was notable on oleic acid, the main fatty acid in almonds, where the ‘Marcona’ allele resulted in an increase of 22 mg per gram of sample.

Finally, two QTLs (*qP-KColor_a1.1* and *qP-KColor_b2.1*) associated with kernel color were mapped on the MC × MI and ‘Marinada’ maps (**Table 2**; **Figure 3**). *qP-KColor_a1.1*, mapped on LG1, explained 25.3% of the PV in MC × MI map, whereas *qP-KColor_b2.1*, found on LG2 was associated with PV of 27.1% in MC × MI (**Table 2**).

## 4 Discussion

The high number of seedlings produced and assessed each year in almond breeding programs highlights the importance of new genomic and phenotypic technologies to enhance breeding efficiency (Font i Forcada et al., 2017). This study examined the genetic basis of several almond quality traits related to kernel physical and chemical characteristics. This research was conducted on a population derived from the cross between the almond cultivars ‘Marcona’ and ‘Marinada’, aiming to improve phenotypic screenings and to provide genetic information that could serve as a foundation for developing DNA markers of breeding interest related to these traits.

Several QTL analyses have been reported in almond using biparental populations, most of them employing a relatively low number of markers (Ballester et al., 1998, 2001; Sánchez-Pérez et al., 2007; Font i Forcada et al., 2012; Fernández i Martí et al., 2013; Goonetilleke et al., 2018; Paizila et al., 2021; Goonetilleke et al., 2023). In this study, we used the recently developed almond Axiom™ 60K SNP array (Duval et al., 2023), which allows the development of highly saturated maps and genome-wide association analyses (Pérez de los Cobos et al., 2023). The marker saturation observed in the maps generated here greatly improved upon that reported in previous linkage maps (Sánchez-Pérez et al., 2007; Font i Forcada et al., 2012; Fernández i Martí et al., 2013; Goonetilleke et al., 2018), demonstrating that the almond 60K SNP array is an excellent tool for genetic analyses in almond. The high-quality linkage map, along with the QTLs reported in this study, will enable the implementation of efficient marker-assisted selection strategies aimed at improving kernel quality traits, which are key almond breeding objectives (Batlle et al., 2017).

High-throughput phenotyping is crucial to increase the efficiency of breeding programs. To this end, standard phenotyping protocols for almond shape traits were compared with image analysis using the newly developed artificial intelligence-based Shape Analyzer software (Jurado, 2024). The strong correlation observed between traits assessed through conventional and image analysis methods for kernel length, width, roundness, and symmetry, along with the discovery of QTLs in identical positions, underscores the reliability of these phenotyping protocols for their routine implementation in breeding programs.

### 4.1 Trait correlations

Most traits phenotyped over multiple years showed significant correlations across those years. This suggests that the phenotypic data obtained is of high quality and that most traits exhibit high heritability. These findings are consistent with other studies on traits like kernel shape (Martínez-Garcia et al., 2019), which facilitates inheritance studies and supports the implementation of marker-based selection approaches.

Identifying correlations between traits can provide valuable insights for more efficient breeding and selection. We found several interesting correlations between physical and chemical traits: for example, kernel weight is correlated with kernel length and width, but these two are not correlated with each other. Additionally, kernel roundness is correlated with kernel length and width, but not with kernel weight. Kernel thickness is correlated with kernel width and shape but not with kernel length, which opens the possibility of discovering kernel shapes different than those of the parents used. Our results indicate that kernel weight is more related to the size than the shape of the kernels, as reported in recent studies (Lipan et al., 2022). High correlations between morphological traits were also found previously in progenies derived from ‘Blanquerna’ × ‘Vivot’ (Fernández i Martí et al., 2013) and ‘Nonpareil’ × ‘Lauranne’ (Goonetilleke et al., 2023). Crack-out percentage and globosity are inversely correlated, and a QTL for both traits has been identified in the same region of LG2. This correlation between these two traits has not been previously reported, and further results are needed to confirm whether this is a common phenomenon in almond or specific to this population. For the chemical kernel composition, it is noteworthy that there is no correlation between total fat content and the composition of fatty acids, but there is a high correlation between most of the different fatty acids, as previously observed (Font i Forcada et al., 2012). Additionally, there is a notable negative correlation between fat and protein content. The highest correlation between physical and chemical traits was observed for the fat content, which was inversely correlated with globosity (-0.45), and for protein content, which is inversely correlated with crack-out percentage (-0.39). These correlations are particularly significant, given that the only detected QTL for these traits was mapped to the same region of chromosome 2. Other interesting correlations of chemical traits have been explored, such as the relationship between oleic acid content and shelf life recently pointed out in rapeseed (Spasibionek et al., 2020) and almond (Sideli et al., 2024).

### 4.2 Genetic analysis of kernel traits

Several QTLs associated with kernel traits were identified by our study, some of which co-localize QTLs previously identified by other authors. For instance, the crack-out percentage QTL (*qP-Crack2.1*), located on chromosome 2, has been reported by previous studies for crack-out and shell hardness (Goonetilleke et al., 2018; Pavan et al., 2021; Sánchez-Pérez et al., 2007; Sideli et al., 2023; Pérez de los Cobos et al., 2023). Both ‘Marcona’ and ‘Marinada’ exhibited hard shells, however wide variation was found in their F_1_ progeny. This suggests the dominance of hard shells and the presence of alleles in heterozygosity in the parental cultivars. The additive effect for crack-out percentage was 6.2% in ‘Marinada’ and 7.3% in ‘Marcona’. Meanwhile, in the MC × MI map, the difference between the highest and lowest mean values was 15.7%, closely matching the combined effect of both parents and supporting this hypothesis. Similarly, for kernel weight, a major QTL was identified at the beginning of LG4, the same chromosome where a previous QTL for this trait was identified in another F_1_ population derived from the cross ‘R1000’ × ‘Desmayo Largueta’ (Sánchez-Pérez et al., 2007) but at a slightly different genomic position (approx. 3 Mbp apart). QTLs for kernel weight have been identified in various genomic regions across studies (Fernández i Martí et al., 2013; Goonetilleke et al., 2023; Pérez de los Cobos et al., 2023), suggesting that kernel weight is a quantitative trait controlled by several genes in the almond germplasm. This was corroborated in our study, where in addition to the main kernel weight QTL (*qP-KWe4.1*), an additional QTL, detected only in the ‘Marcona’ map, indicated the presence of multiple alleles associated with weight variation in these cultivars. Additionally, a QTL for kernel width (*qP-KWidth_SA4.1*) was also identified, overlapping with *qP-We4.1*, suggesting that width may influence the observed differences in fruit weight. These interactions and the numerous QTLs reported for this trait highlight the challenges in implementing effective breeding strategies for weight gain in breeding programs.

QTLs for various kernel morphology traits, including length, thickness, width, roundness, symmetry, and shoulder angle were identified at various genomic locations. Notably, major QTLs for kernel length and width were found 25 Mbps apart on chromosome 1, suggesting that different genetic mechanisms control these traits. *qP-KLen1.1*, associated with kernel length, had previously been mapped in a panel of 98 almond cultivars (Font i Forcada et al., 2015a) and in an F_1_ population (‘Vivot’ × ‘Blanquerna’) (Fernández i Martí et al., 2013). Additionally, Goonetilleke et al. (2023) reported two overlapping QTLs for kernel width and length on LG1 of the ‘Lauranne’ map. However, this region is situated between the kernel length and width QTLs identified in the MC × MI population, suggesting that *qP-KWidth1.1* is a new QTL associated with kernel width found in almonds. For roundness and symmetry, QTLs for the same traits were recently reported in the same genetic region of chromosome 1 (Goonetilleke et al., 2023). A QTL for kernel thickness was also identified on LG4 (*qP-KThick4.1*). Finally, another QTL for roundness was mapped on LG7 in a different position where a QTL for sphericity was reported on the same LG in a ‘Nonpareil’ × ‘Lauranne’ population (Goonetilleke et al., 2023).

In this population, we studied the segregation of the shoulder character, which is being analyzed for the first time in this study. We observed a strong correlation between kernel shoulder, width, and thickness, with a negative correlation with kernel length. Similar to the pattern seen with shape QTLs, QTLs associated with kernel shoulder exhibit a broader confidence interval compared to those for length or width, and similar to shape–related traits. This suggests that the QTL for kernel shoulder is likely influenced by both kernel length and width QTLs. To further validate QTLs for kernel length and width, it would be preferable to use a population where the kernel shoulder is not segregating.

Manual measurements for kernel shape traits and image analysis using artificial intelligence were employed in this study for QTL mapping. Despite the high correlation between both sets of phenotypic data, some QTLs identified through image analysis were not detected with manual measurements. This highlights the efficiency of using this new phenotyping method, not only in reducing data collection efforts but also in increasing the identification of new QTLs.

### 4.3 Genetic analysis of kernel chemical composition

A cluster of overlapping QTLs located at the lower end of LG1 was identified for the fatty acids. These results indicate that a single molecular marker could predict the content of all these fatty acids. This is particularly significant given the prevalence of these acids in almonds, their role in health benefits associated with almond consumption, and their impact on kernel rancidity, which compromises flavor and postharvest shelf life (Barreca et al., 2020; Becerra-Tomás et al., 2019; Flankin and Mitchell, 2019). Although, these fatty acids were quantified in only one year; the fact that all acids mapped to the same region of LG1, along with the high percentage of variance explained by these QTLs (over 25% for most of the acids), underscores the reliability of the results despite validation with additional year data. Moreover, this region coincides with a QTL previously documented in almonds associated with fatty acids content and located at the bottom of LG1 in a biparental population (‘Vivot’ × ‘Blanquerna’) and a panel of 98 almond accessions (Font i Forcada et al., 2012, 2015b; Sideli et al., 2024). Additionally, this region of LG1 encompasses markers recently associated with different degrees of rancidity (Sideli et al., 2024). It is known that fatty acids and phenolic compounds are related to kernel rancidity, so the overlap of these two regions highlights the impact of these acids on controlling rancidity levels. Furthermore, the additive effect observed with these acids (up to 28.7 mg/g kernel weight for oleic acid) underscores the significant potential for improvement through breeding, which could result in new cultivars with an enhanced fatty acid profile.

We identified four QTLs associated with protein content on LGs 2, 3, 5, and 7. Among these, only *qP-Protein3.1* and *qP-Protein7.1* co-localize with QTLs mapped by previous studies (Font i Forcada et al., 2012, 2015b). However, the primary QTL for protein content discovered in this study, *qP-Protein2.1*, did not align with any previously identified QTL region, suggesting that the regulation of protein content involves multiple genes. For fiber and fat content, QTLs related to these traits are reported for the first time by our study. Both *qP-Fiber3.1* and *qP-Fat3.1* showed low significance, which could be related to their dependence on environmental conditions, as different stresses like drought and temperature have been linked to interannual variation in these compounds (Kodad et al., 2018). This is consistent with the low proportion of phenotypic variability explained by cultivars for fiber (15.7%) and fat (28.4%) contents in previous studies (Romero et al., 2011), highlighting the low heritability of these compounds.

## 5 Conclusions

In this study, several kernel quality and chemical traits were investigated in a segregating almond F_1_ population, followed by QTL analyses. The use of the first high-density SNP array developed in almond (Axiom™ 60K SNP array), combined with traditional phenotyping protocols for kernel shape traits with image analysis using artificial intelligence, enhanced the detection power of QTLs. Notably, a region at the lower end of LG1 was mapped where QTLs for different fatty acids co-localized. Additionally, major QTLs for kernel shape and dimensions were identified in a few genomic regions. The results revealed multiple QTLs distributed across the entire genome, with QTL hotspots that can be used by breeders to further implement marker-assisted breeding in almond. These regions will be targeted for further fine mapping and *in silico* gene annotation to identify genes and polymorphisms associated with the phenotypic variation of these QTLs.

## Data Availability

The original contributions presented in the study are included in the article/**Supplementary Material**. Further inquiries can be directed to the corresponding author.

## Supplementary material

Supplementary Table 1 Information on the SSR markers used in this study for map construction.

Supplementary Table 2 Phenotypic values of ‘Marcona’ and ‘Marinada’ parentals, number of individuals considered for QTL analyses (N), and minimum (Min), maximum (Max), mean, and standard deviation (SD) values of the ‘Marcona’ × ‘Marinada’ population. The results of the Shapiro-Wilk normality tests are also included.

Supplementary Table 3 Coefficient of determination (R^2^) for all traits for which LSmean values were calculated.

Supplementary Table 4 Pairwise correlation coefficient among phenotyped traits. Significant (p<0.001) Spearman correlation coefficients are highlighted in bold.

Supplementary Table 5 Summary of marker position on the ‘Marcona’, ‘Marinada’, and ‘Marcona’ × ‘Marinada’ linkage maps.

Supplementary Table 6 Summary of all detected QTLs.
