# Supplementary material for: QTL mapping of almond kernel quality traits in the F1 progeny of ‘Marcona’ × ‘Marinada’
Source: Front Plant Sci. 2024 Nov 27;15:1504198. doi: 10.3389/fpls.2024.1504198 (PMC11631582; doi:10.3389/fpls.2024.1504198)
Supplement: Supplementary Table 2 — Phenotypic values of ‘Marcona’ and ‘Marinada’ parentals, number of individuals considered for QTL analyses (N), and minimum (Min), maximum (Max), mean, and standard deviation (SD) values of the ‘Marcona’ × ‘Marinada’ population. The results of the Shapiro-Wilk normality tests are also included. [file Table2.docx]

**Supplementary Table 2.** Phenotypic values of ‘Marcona’ and ‘Marinada’ parentals, number of individuals considered for QTL analyses (N), and minimum (Min), maximum (Max), mean, and standard deviation (SD) values of the ‘Marcona’ × ‘Marinada’ population. The results of the Shapiro-Wilk normality tests are also included.

|  |  |  |  | **Marcona × Marinada** | | | | **Shapiro-Wilk test** | |
| --- | --- | --- | --- | --- | --- | --- | --- | --- | --- |
| **Trait** | **Marcona** | **Marinada** | **N** | **Min** | **Max** | **Mean** | **SD** | **W** | ***p*-Value** |
| Kernel weight (g) | 1.52 | 1.15 | 87 | 0.89 | 1.61 | 1.28 | 0.14 | 0.987 | 0.514 |
| Crackout (%) | 25.55 | 31.14 | 87 | 22.36 | 56.90 | 30.00 | 7.53 | 0.871 | <0.001 |
| Kernel length (mm) | 21.41 | 22.58 | 87 | 16.77 | 24.87 | 21.32 | 1.69 | 0.993 | 0.945 |
| Kernel length SA (mm) | - | - | 87 | 16.84 | 25.84 | 21.24 | 1.75 | 0.994 | 0.984 |
| Kernel width (mm) | 17.23 | 14.01 | 87 | 12.17 | 17.95 | 15.16 | 1.11 | 0.987 | 0.532 |
| Kernel width SA (mm) | - | - | 87 | 11.71 | 18.67 | 15.70 | 1.28 | 0.984 | 0.369 |
| Kernel thickness (mm) | 9.20 | 8.52 | 87 | 7.21 | 11.56 | 9.03 | 0.90 | 0.987 | 0.521 |
| Kernel roundness | 1.24 | 1.61 | 87 | 0.56 | 0.84 | 0.71 | 0.07 | 0.974 | 0.076 |
| Kernel roundness SA | - | - | 86 | 0.84 | 0.94 | 0.91 | 0.02 | 0.917 | <0.001 |
| Kernel globosity | 1.78 | 1.65 | 87 | 1.31 | 2.27 | 1.65 | 0.16 | 0.963 | 0.015 |
| Kernel symmetry (Jaccard) | - | - | 86 | 0.65 | 0.93 | 0.88 | 0.06 | 0.854 | <0.001 |
| Kernel symmetry (SSIM) | - | - | 86 | 0.95 | 0.98 | 0.97 | 0.01 | 0.929 | <0.001 |
| Kernel shoulder | - | - | 87 | 1.00 | 5.00 | 2.00 | 1.13 | 0.843 | <0.001 |
| Kernel shoulder angle | - | - | 85 | 7.41 | 44.18 | 27.36 | 8.12 | 0.986 | 0.501 |
| Tegument Color L* | 24.30 | 26.59 | 87 | 22.62 | 32.61 | 26.13 | 2.19 | 0.947 | <0.001 |
| Tegument Color a* | 7.72 | 11.52 | 87 | 7.83 | 11.12 | 9.79 | 0.68 | 0.981 | 0.223 |
| Tegument Color b* | 13.79 | 20.39 | 87 | 14.07 | 20.05 | 17.41 | 1.36 | 0.987 | 0.561 |
| Kernel Color L* | 48.63 | 45.63 | 87 | 42.83 | 49.20 | 46.20 | 1.48 | 0.986 | 0.505 |
| Kernel Color a* | -0.75 | -1.17 | 87 | -1.54 | -0.59 | -1.03 | 0.20 | 0.986 | 0.497 |
| Kernel Color b* | 10.61 | 12.10 | 87 | 10.34 | 13.00 | 11.71 | 0.60 | 0.990 | 0.753 |
| Protein (%) | 27.31 | 23.37 | 87 | 18.47 | 27.72 | 24.80 | 1.79 | 0.949 | <0.001 |
| Fiber (%) | 5.48 | 9.24 | 87 | 2.80 | 7.56 | 4.88 | 0.94 | 0.975 | 0.094 |
| Fat (%) | 55.03 | 52.08 | 83 | 46.62 | 58.83 | 53.01 | 2.66 | 0.986 | 0.552 |
| Myrisitc acid (mg/g) | 0.03 | 0.03 | 72 | 0.03 | 0.08 | 0.05 | 0.01 | 0.953 | <0.001 |
| Palmitic acid (mg/g) | 6.92 | 7.30 | 72 | 6.78 | 12.84 | 9.03 | 1.45 | 0.950 | <0.001 |
| Palmitoleic acid (mg/g) | 0.70 | 0.51 | 72 | 0.37 | 1.32 | 0.75 | 0.20 | 0.974 | 0.146 |
| Margaric acid (mg/g) | 0.07 | 0.06 | 72 | 0.06 | 0.14 | 0.09 | 0.02 | 0.962 | 0.029 |
| cis-10-Heptadecenoic acid (mg/g) | 0.13 | 0.14 | 72 | 0.10 | 0.82 | 0.17 | 0.09 | 0.507 | <0.001 |
| Stearic acid (mg/g) | 1.64 | 1.29 | 69 | 1.03 | 3.45 | 2.08 | 0.55 | 0.972 | 0.126 |
| Oleic acid (mg/g) | 81.60 | 80.94 | 72 | 52.90 | 160.85 | 99.76 | 28.21 | 0.955 | 0.012 |
| Vaccenic acid (mg/g) | 1.70 | 2.27 | 69 | 1.83 | 3.15 | 2.44 | 0.28 | 0.984 | 0.540 |
| Linoelaidic acid (mg/g) | 0.03 | 0.03 | 56 | 0.03 | 0.19 | 0.04 | 0.03 | 0.564 | <0.001 |
| Linolenic acid (mg/g) | 6.62 | 6.87 | 72 | 5.33 | 17.12 | 8.80 | 2.56 | 0.936 | <0.001 |
| Arachidic acid (mg/g) | 0.07 | 0.09 | 71 | 0.07 | 0.19 | 0.11 | 0.03 | 0.944 | <0.001 |
| cis-11-eicosenoic acid (mg/g) | 0.07 | 0.10 | 72 | 0.06 | 3.10 | 0.09 | 0.35 | 0.137 | <0.001 |
| cis-11.14-eicosadienoic acid (mg/g) | 0.33 | 0.30 | 57 | 0.07 | 7.30 | 0.21 | 1.10 | 0.290 | <0.001 |
