# Supplementary material for: QTL mapping of almond kernel quality traits in the F1 progeny of ‘Marcona’ × ‘Marinada’
Source: Front Plant Sci. 2024 Nov 27;15:1504198. doi: 10.3389/fpls.2024.1504198 (PMC11631582; doi:10.3389/fpls.2024.1504198)
Supplement: Supplementary Table 3 — Coefficient of determination (R2) for all traits for which LSmean values were calculated. [file Table3.docx]

**Supplementary Table 3.** Coefficient of determination (R^2^) for all traits for which LSmean values were calculated.

| **Trait** | **R^2^** |
| --- | --- |
| Kernel weight | 0.71 |
| Kernel length | 0.64 |
| Kernel length SA | 0.85 |
| Kernel width | 0.74 |
| Kernel width SA | 0.80 |
| Kernel thickness | 0.60 |
| Kernel roundness | 0.77 |
| Kernel roundness SA | 0.91 |
| Kernel globosity | 0.63 |
| Kernel symmetry (Jaccard) | 0.91 |
| Kernel symmetry (SSIM) | 0.88 |
| Crack-out | 0.80 |
| Tegument color L* | 0.47 |
| Tegument color a* | 0.74 |
| Tegument color b* | 0.78 |
| Kernel color L* | 0.66 |
| Kernel color a* | 0.65 |
| Kernel color b* | 0.74 |
| Protein content | 0.65 |
| Fiber content | 0.45 |
